# The effectiveness and safety of Chinese herbal formulas on skin photoaging: A protocol for systematic review and meta-analysis

**DOI:** 10.1097/MD.0000000000024197

**Published:** 2021-01-22

**Authors:** Ji-Yong He, Xiao-Yv Yv, Jing-Dong Wu, Ling Lv, Xiao-Qing Zhang, Tie-Ming Ma, Yu Zhang

**Affiliations:** a Graduate School, Liaoning University of Traditional Chinese Medicine; b Affiliated Hospital of Liaoning University of Traditional Chinese Medicine; c Liaoning University of Traditional Chinese Medicine Hospital 2nd Affiliated Hospital; d Liaoning University of Traditional Chinese Medicine, Shenyang, Liaoning, China.

**Keywords:** Chinese herb formula, photoaging, protocol, systematic review

## Abstract

**Background:**

Skin photoaging (SP) is a complex and complicated process of skin characteristic changes caused by excessive sunlight. Wrinkles, looseness, coarseness, and increase or loss of pigment are the main clinical manifestations of the disease. The pathogenesis of SP mainly involving oxidative stress, inflammatory reaction, immune dysregulation and DNA damage, and so on. In recent years, traditional Chinese medicine, as an significant form of complementary and alternative medicine, has attracted the more and more attention within the field of health care and indicated a desirable effect on SP. Chinese herbal formula (CHF) is an essential part of traditional Chinese medicine interventions, and the number of clinical trails on SP treated by CHFs have shown a growing trend. Therefore, we developed this systematic review and meta-analysis protocol to assess the effectiveness and safety of CHFs in the therapy of SP, so as to provide reliable evidence-based evidence for clinical decision-making.

**Methods:**

A overall literature retrieval will be carried out in 9 electronic journal database. We will include randomized controlled trials (RCTs) on CHFs alone or combined with routine western medicine measures in the treatment of SP. The outcomes we focused on are consists of symptom score (skin relaxation, telangiectasia, pore coarseness, pigmentation, etc), total effective rate, and adverse reactions. Meta-analysis will be performed using Stata 13.0 software. Literature retrieval and screening, data extraction, risk of bias assessment of RCTs, evidence confidence rating by grading of recommendations assessment, development, and evaluation method and methodological quality assessment of systematic review by assessment of multiple systematic reviews-2 will be conducted independently by 2 reviewers, and disagreements will be resolved through discussion or judged by a third senior reviewer.

**Results:**

This systematic review and meta-analysis will pool the proof of RCTs on SP treated by CHFs alone or combined with conventional western medicine treatments. The findings of this study will be presented at relevant conferences and submitted to peer-reviewed journals for publication.

**Conclusion:**

We expect that the results of this systematic review will provide comprehensive and reliable evidence for clinicians and policy makers.

**Registration number:**

INPLASY 2020120005

## 1 Introduction

Skin photoaging (SP) is a chronic inflammatory process induced by sunlight, especially ultraviolet radiation.^[1]^ The main clinical manifestations were wrinkles, pigmentation, or depigmentation, telangiectasia, rough, dry, and flabby skin. The aging degree mainly depends on the exposure to sunlight and the content of skin pigment.^[2]^

The pathogenesis of SP are complex and complicated, mainly involving oxidative stress, inflammatory reaction, apoptosis, immune imbalance, collagen repair, and so on. The specific mechanisms are as follows:

(1) Oxidative stress: ultraviolet radiation for a long time can produce excessive reactive oxygen species and reactive oxygen radicals, which directly or indirectly lead to the damage of proteins, lipids, and nucleic acids in cells, activate nuclear factor-κ-gene binding (NF-κB), and mitogen-activated protein kinase (MAPK) signaling pathways, upregulate matrix metalloproteinase (MMPs), including MMP-1, MMP-3, and MMP-9, promote collagen degradation, and also cause cells to degrade collagen apoptosis.^[3]^
(2) Apoptosis: long time ultraviolet irradiation may activate FAS/FasL signal transduction pathway and mitochondrial pathway, which leads to the cleavage of the corresponding caspase downstream, causing cascade reaction and eventually causing cell apoptosis.^[4,5]^
(3) Collagen repair: ultraviolet radiation tend to increase the transcription and expression of MMPs and promote the degradation of collagen by activating MAPK pathway, increasing the concentration of oxidized glutathione, activating cjun N-terminal kinase and p38 MAPK pathway.^[6,7]^
(4) Inflammatory reaction: ultraviolet irradiation may promote the synthesis and release of various inflammatory mediators, induce the phosphorylation of extracellular regulated protein kinases (ERK) pathway, and induce the overexpression of tumor necrosis factor (TNF)-α, which can bring about inflammatory injury, increase the transcription and expression of MMPs, and promote the degradation of collagen.^[8,9]^
(5) Ultraviolet irradiation tend to produce a large number of oxygen free radicals, and lead to the decrease of hyaluronic acid synthase expression, skin water content, causing skin aging.^[10,11]^

Oxygen free radicals can cause immune dysfunction, which in turn give rise to obstacles in the scavenging of oxygen free radicals, forming a vicious cycle. Furthermore, it can also destroy the morphology and structure of epidermal langerhans cells, reduce immunity, and damage skin health.^[12]^

Therefore, the mechanisms of SP are multiple, and the protection against SP is particularly necessary. Protection for SP generally refers to sun protection through sunshade umbrellas, wearing sunscreen caps and sunglasses, and applying sunscreen, and so on.^[13]^ The treatment of SP mainly includes retinoic acid drugs, antioxidants, ultra-pulsed CO2 lattice laser, intense pulsed light, 5-aminolevulinic acid photodynamic therapy, botulinum toxin injection, hyaluronic acid filling, and chemical exfoliation therapy, and so on.^[14]^ However, their therapeutic effects remain to be further verified.^[15]^

Although traditional Chinese medicine is not the mainstream treatment for skin diseases, it is gradually accepted as an irreplaceable form of complementary and alternative medicine in western countries.^[16]^ Many Chinese medicines and their extracts have shown good therapeutic effects on SP.^[17]^ Recent works have shown that paeonol can regulate inflammatory factors (interleukin [IL]-6, TNF-α) and inhibit inflammatory reaction; improve the protein content of JNKs, H2AX, TOPK, and p38 through p38MAPK and JNKs signaling pathway.^[18]^ Gypenosides can inhibit the secretion of inflammatory factors (IL-6, TNF-α, IL-1β) from HaCaT cells damaged by light. Salvianolic acid B can reduce hydroxyproline content, superoxide dismutase activity, and the formation of free radical metabolites.^[19]^*Lycium barbarum* polysaccharide can inhibit the expression of MMPs (MMP-1, MMP-3, and MMP-9) in fibroblasts and alleviate SP.^[20]^ Yu Ping Feng powder can repair collagen fiber and elastic fiber, reduce the content of keratin (CK5, CK14).^[21]^ Sha Shen Mai Dong decoction can enhance the antioxidation ability of skin, increase the activity of Glutathione peroxidase, and Superoxide dismutase, enhance the free radical scavenging ability, and reduce the damage.^[22]^ Shi Jing pill can reduce the content of MMP-1 and increase the content of tissue inhibitor of metalloproteinases-1, so as to alleviate the damage of embryonic skin fibroblasts-1 cells caused by light radiation.^[23]^ Tao Hong Si Wu Decoction can reduce the serum inflammation (IL-1, TNF-α), inhibit the gene expression of MMP-1 and MMP-3 in skin tissue, and alleviate the damage of ultraviolet radiation on skin.^[24]^

Chinese herbal formulas (CHFs) is an significant part of traditional Chinese medicine (TCM) interventions. In recent years, it has shown a trend of gradual increase in the number of randomized controlled trials (RCTs) of CHFs alone or in combination with conventional western medicine therapy for SP. However, no systematic review summarize these results of clinical studies have not been retrieved.

## 2 Objective

This systematic review and meta-analysis aims to pool these proofs of RCTs, and then assess the effectiveness and safety of CHFs on SP.

## 3 Methods

### 3.1 Study protocol and registration

This protocol was designed in accordance with Cochrane Handbook for Systematic Reviews of Interventions and Preferred Reporting Items for Systematic Reviews and Meta-Analyses Protocols (PRISMA-P) 2015 checklist.^[25]^ If necessary, we will describe any changes in the systematic review. This research has been registered on the International Platform of Registered Systematic Review and Meta-analysis Protocols (INPLASY no. 2020120005, https://inplasy.com/?s=2020120005)

### 3.2 Eligibility criteria

#### 3.2.1 Types of reviews

RCTs are eligible for inclusion which should assess at least 1 outcome. Quasi-RCTs, literature review, duplicated publications, case report, animal experiments, editorials, and pharmaceutical experiments will be excluded.

#### 3.2.2 Participants

Patients diagnosed as SP by any guidelines or expert consensus with clear diagnostic criteria will be included, regardless of age, region, race, or country.

#### 3.2.3 Interventions and comparisons

CHFs alone or combined with routine western medicine interventions will meet the eligibility criteria in the treatment group. Conventional interventions in western medicine or no treatment will be applied in the control group, which include retinoids, antioxidants, ultra-pulsed CO2 dot array laser, intense pulsed light, and so on. There will be no limit for CHFs on the dosage, form, frequency, and duration of treatment. The same conventional measures of western medicine must be used in the comparator arm.

#### 3.2.4 Outcomes

The primary outcome we focused on contain symptom scores (degree of skin relaxation, degree of telangiectasia, degree of pore coarseness, degree of pigmentation, etc), total effective rate, and adverse reactions. There were no plan for secondary outcome evaluation in this study.

### 3.3 Information sources

An all-round retrieval will be performed in 9 electronic journal databases from their inception to November 2020, which comprise Chinese National Knowledge Infrastructure, Chinese Biomedical Database, Chongqing VIP information, Wanfang database, PubMed, EMBASE, Web of Science, Cochrane Central Register of Controlled Trials, and The Cumulative Index to Nursing & Allied Health Literature. We will also search the following databases to identify clinical trials being in progress or completed: Clinical Trials.gov trials registry, the World Health Organization clinical trials registry, and Chinese Clinical Trial Registry. Furthermore, bibliography of identified articles and grey literature will also be retrieved to avoid omissions.^[26]^ The language of documents retrieved are limited to Chinese and English. The search strategy of Pubmed is as follows. Besides, we will adjust it on the basis of the characteristics of each databases.

**Table:** 

**Search Strategy in Pubmed**(“skin aging”[MeSH Terms] OR “photoaging” [Title/Abstract] OR “light aging” [Title/Abstract]) AND (“medicine, chinese traditional” [MeSH Terms] OR“chinese herbal” [Title/Abstract] OR “chinese medic∗” [Title/Abstract] OR “herb∗” [Title/Abstract] OR “decoction” [Title/Abstract] OR “chinese patent medicine” [Title/Abstract] OR “traditional chinese medicine prescription” [Title/Abstract]) AND (“randomized controlled trial” [Filter] OR“randomized” [Title/Abstract] OR “randomly” [Title/Abstract] OR “meta analy∗” [Title/Abstract])

### 3.4 Studies selection

**Figure 1:**
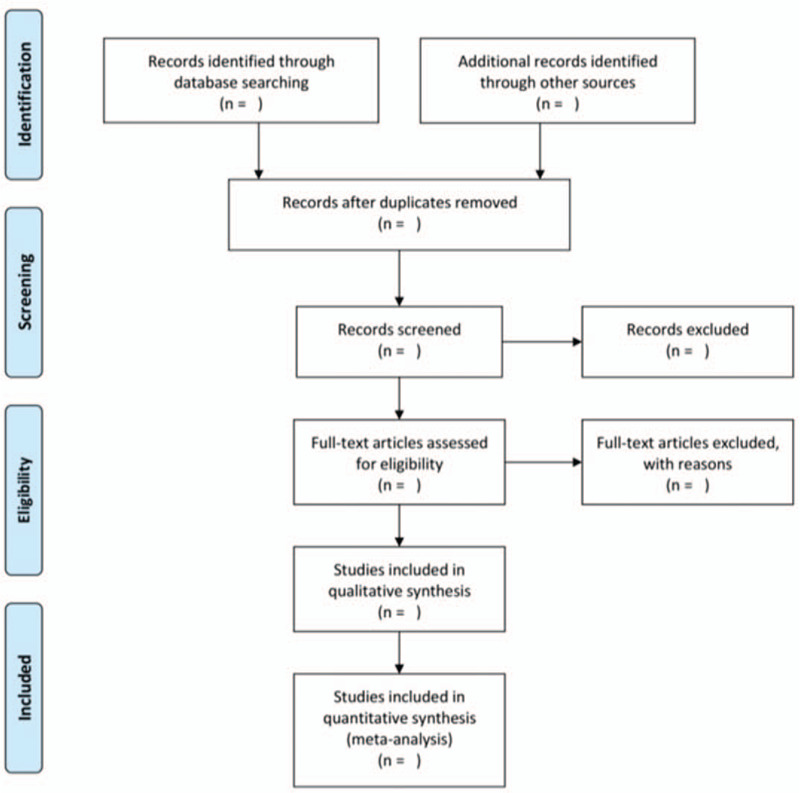
Flow diagram of the literature searching and study selection. From: Moher D, Liberati A, Tetzlaff J, Altman DG, The PRISMA Group (2009). Preferred Reporting Items for Systematic Reviews and Meta-Analyses: The PRISMA Statement. PLoS Med 6(6): e1000097. doi:10.1371/journal.pmed1000097.

All retrieved literature will be imported into Note Express V3.0 software and duplicate literature will be eliminated. In the first instance, 2 researchers (He JJ and Lv L) will independently and respectively screened the included studies on the basis of the inclusion criteria, and then download the remaining studies for further screening by reading full texts. If any disagreement arises in the process, it will be decided through discussion or by a third senior researcher (Wu JD). Two researchers will take notes all excluded literature and provide reasonable reasons for exclusion.^[27]^ Details of the selection process will be presented in the PRISMA flow chart (Fig. 1).

### 3.5 Data extraction

The key information of the included trails will be extracted by 2 independent reviewers (He JJ and Lv L) in accordance with the pre-designed form. The final decisions will be made by consensus process and disagreements will be adjudicated by a third high-level reviewer (Wu JD). Epidata 3.1 software (The EpiData Association, Odense, Denmark, 2003–2008) will be applied to extract data and check the consistency of data. We will collect the following information from each clinical trails included: the first author, publication year, primary locality of the study, sample size (research group/control group), outcomes, range of age (research group/control group), gender distribution (male/female), diagnostic criteria, and funding. If any material information elements are missing, we will attempt to contact the authors for the desired data. We will employ intention-to-treat analysis in case of missing data are unobtainable. Sensitivity analysis will also be executed to address the potential impact of missing data, which will be discussed if necessary.^[28,29]^

### 3.6 Risk of bias assessment

Two investigators (He JJ and Lv L) will assess the methodological quality of the RCTs separately and individually by risk of bias 2.0 (RoB 2.0) tool recommended by Cochrane handbook.^[30]^ This tool can be applied to evaluate the possibility and sources of bias in RCTs comprehensively from 5 areas, which are “risk of bias arising from the randomization process,” “risk of bias due to deviations from the intended interventions,” “missing outcome data,” “risk of bias in measurement of the outcome,” and “risk of bias in selection of the reported result.” For several signaling questions set up by each module, the investigators should respond to “Yes,” “Probably yes,” “Probably no,” “No (not),” “Not applicable” or “No information (unclear),” respectively.^[31]^ If any divergence arises during this procedure, the 2 investigators will reach agreement through discussion or be adjudicated by a third senior investigator (Wu JD).

### 3.7 Statistical analysis

#### 3.7.1 Meta-analysis

We will perform meta-analysis using Stata 13.1 software (Stata-Corp LP, College Station, TX 77845). If available data are insufficient, a descriptive analysis will be carried out.^[32]^ The *Q*-test and *I*^2^ values will be used to indicate inter-study heterogeneity. When the *P*-value of *Q*-test > 0.1 and *I*^2^ < 50%, a fixed-effects model was applied; otherwise, a random-effects model was used. Binary variables were expressed by odds ratio with 95% confidence interval, and continuous variables by mean difference with 95% confidence interval. If significant heterogeneity is found, we will try to explore the source of heterogeneity by subgroup analysis based on specified effect modifiers as follows: interventions, publication year, dosage form, participant's average age, sample size, treatment duration, publication language, and so on.^[33]^

#### 3.7.2 Sensitivity analysis

Studies included with different levels of methodological quality tend to affect the results of pooled effect estimates. Therefore, we will evaluate the robustness of meta-analysis results by eliminating low-quality studies for sensitivity analysis based on the assessment results of RoB2.0.^[34]^ In addition, we will judge the stability of parameter estimation results by permutation effect model.

#### 3.7.3 Publication bias

Publication bias will be assessed adopting qualitative and quantitative methods. We will use the graphical method of inverted funnel plot to judge potential publication bias qualitatively. Egger regression test will be used for continuous data, and Peter for dichotomies data to assess the publication bias quantitatively, if the studies included are sufficient (n ≥ 10).^[35]^

### 3.8 Assessment of quality of evidence

Grading of recommendations assessment, development, and evaluation (GRADE) system will be adopted to assign levels for meta-analysis evidence. The confidence of evidence pooled within the systematic review and meta-analysis will be appraised independently and separately by 2 review authors (He JJ and Lv L). GRADE is not only a rating system for proofs, it also summarizes evidence for systematic reviews and guidelines in the field of health care and provides a transparent, reliable, and structured method.^[36]^ RCT is initially identified as high-quality proof. Study limitations/risk of bias, publication bias, imprecision, inconsistency and indirectness are 5 factors that may degrade the confidence of evidence quality. The overall quality of evidence will be defined as “high,” “moderate,” “low,” or “very low.” Any disagreement during this process will be reached by 2 investigators through consultation or adjudicated by a third senior investigator (Wu JD). Kappa statistics will be calculated to understand the consistency of classification by GRADE method between 2 authors.^[37]^

### 3.9 Report of systematic review

This systematic review and meta-analysis will be reported following Preferred Reporting Items for Systematic Reviews and Meta-Analyses Extension for Chinese Herbal Medicines 2020 (PRISMA-CHM 2020) checklist Item.^[38]^

### 3.10 Assessment of methodological quality

Assessment of multiple systematic reviews-2 (AMSTAR-2) is a practical, reliable, and effective tool for measuring the methodological quality of systematic review.^[39]^ It includes 16 items involving the whole process of topic selection, design, registration, data extraction, data statistical analysis, and discussion of systematic review.^[40]^ Two investigators (He JJ and Lv L) will use the AMSTAR-2 tool to evaluate the overall quality of the systematic review and meta-analysis by item based on its guidance documents, during which disagreements will be adjudicated through discussion or by a third high-level investigator (Wu JD). In this research, scores will be completed and calculated through the online AMSTAR-2 checklist (https://amstar.ca/Amstar_Checklist.php). The overall methodological quality of systematic review may be classified as “high,” “moderate,” “low,” and “very low.” We will calculate kappa statistics to make clear the consistency of evaluation by AMSTAR-2 between 2 evaluators.

### 3.11 Strengths and limitations of this study

(1) This study will be the first systematic review and meta-analysis to synthesize the data of clinical trails on the treatment of SP by CHFs alone or in combination with conventional western medicine measures.
(2) We will use a variety of tools to formally assess the risk of bias of RCTs, the confidence of proof for meta-analysis, and the methodological and reporting quality of systematic reviews.
(3) The language of literature retrieval is limited to Chinese and English, which may lead to omission.

## 4 Discussion

CHFs are typical representative of TCM interventions, and has been put into clinical practice for thousands of years in China.^[41]^ As an essential form of ccomplementary and alternative therapies, it has been widely used in the world, because of its curative effect and safety.^[42]^ CHFs are formulated on the basis of TCM theory and syndrome differentiation, which are composed of a variety of single traditional Chinese medicine mainly originating from natural sources, including plants, animals, minerals, and some chemical or biological products.^[43]^

The mechanism of SP are complex and multiple, and sufficient evidence have shown it is closely associated with oxidative stress, inflammatory response, apoptosis, immune dysregulation, collagen repair. Many kinds of Chinese medicinal herbs and herbal extracts have been authenticated the effects on regulating cytokines, signal transduction pathways, and oxidative stress and immunity, and so on for SP.^[44]^ Owing to the diversity of active ingredients in CHFs and the potential synergistic therapeutic effects, which can make them have a wide range of targets and multiple therapeutic mechanisms.^[45]^

Therefore, it is necessary to formulate this systematic review and meta-analysis to synthesize these accessible clinical evidence, and we hope this systematic review will provide more comprehensive, reliable, and practical evidence for clinical decision-making and further research.

## Acknowledgments

This research is completed in the Key Laboratory of acupuncture, moxibustion, health preservation and rehabilitation of Liaoning province.

## Author contributions

**Conceptualization:** Jiyong He, Xiao-Yv Yv, Xiao-Qing Zhang, Tie-Ming Ma.

**Data curation:** Xiao-Qing Zhang.

**Formal analysis:** Jiyong He, Yu Zhang.

**Funding acquisition:** Ling Lv.

**Investigation:** Jiyong He, Yu Zhang.

**Methodology:** Jiyong He, Jing-Dong Wu.

**Project administration:** Jiyong He, Ling Lv.

**Resources:** Ling Lv, Yu Zhang.

**Software:** Jiyong He.

**Supervision:** Jing-Dong Wu.

**Validation:** Jing-Dong Wu.

**Writing – original draft:** Xiao-Yv Yv, Jiyong He, Tie-Ming Ma.

**Writing – review & editing:** Xiao-Yv Yv, Jiyong He, Jing-Dong Wu, Tie-Ming Ma, Xiao-Qing Zhang.
